# Gene co-expression network analysis identifies hub genes associated with different tolerance under calcium deficiency in two peanut cultivars

**DOI:** 10.1186/s12864-023-09436-9

**Published:** 2023-07-27

**Authors:** Kang Tang, Lin Li, Bowen Zhang, Wei Zhang, Ningbo Zeng, Hao Zhang, Dengwang Liu, Zinan Luo

**Affiliations:** 1 College of Agriculture, Hunan Agricultural University, No. 1 Nongda Road, Changsha, 410128 Hunan China; 2 Arid Land Crop Research Institute, Hunan Agricultural University, No. 1 Nongda Road, Changsha, 410128 Hunan China; 3 Hunan Peanut Engineering & Technology Research Center, No. 1 Nongda Road, Changsha, 410128 Hunan China; 4 College of Plant Protection, Hunan Agricultural University, No.1 Nongda Road, Changsha, 410128 Hunan China

**Keywords:** Peanut, Transcriptome, Weighted gene co-expression network analysis (WGCNA), Differentially expressed genes (DEGs), Hub genes

## Abstract

**Background:**

Peanut is an economically-important oilseed crop and needs a large amount of calcium for its normal growth and development. Calcium deficiency usually leads to embryo abortion and subsequent abnormal pod development. Different tolerance to calcium deficiency has been observed between different cultivars, especially between large and small-seed cultivars.

**Results:**

In order to figure out different molecular mechanisms in defensive responses between two cultivars, we treated a sensitive (large-seed) and a tolerant (small-seed) cultivar with different calcium levels. The transcriptome analysis identified a total of 58 and 61 differentially expressed genes (DEGs) within small-seed and large-seed peanut groups under different calcium treatments, and these DEGs were entirely covered by gene modules obtained via weighted gene co-expression network analysis (WGCNA). KEGG enrichment analysis showed that the blue-module genes in the large-seed cultivar were mainly enriched in plant-pathogen attack, phenolic metabolism and MAPK signaling pathway, while the green-module genes in the small-seed cultivar were mainly enriched in lipid metabolism including glycerolipid and glycerophospholipid metabolisms. By integrating DEGs with WGCNA, a total of eight hub-DEGs were finally identified, suggesting that the large-seed cultivar concentrated more on plant defensive responses and antioxidant activities under calcium deficiency, while the small-seed cultivar mainly focused on maintaining membrane features to enable normal photosynthesis and signal transduction.

**Conclusion:**

The identified hub genes might give a clue for future gene validation and molecular breeding to improve peanut survivability under calcium deficiency.

**Supplementary Information:**

The online version contains supplementary material available at 10.1186/s12864-023-09436-9.

## Background

Cultivated peanut (*Arachis hypogaea* L.) is an allotetraploid (2n = 4x = 40) originated from South America [1]. The genome is composed of A and B sub-genomes, which were derived from the hybridization of two ancient diploid species, *A. duranensis* (A-genome) and *A. ipaënsis* (B-genome), followed by chromosome duplication [1]. Peanut is one of the major protein and vegetable oil resources grown worldwide and needs relatively low fertilizer input. China, India, Nigeria and USA are the four largest peanut producers in the world [2].

Ca^2+^ is an essential plant nutrient that plays an important role in plant growth and development processes such as cell division, cell polarity, circadian rhythms, stomatal closure, senescence, and in response to various abiotic stresses [3]. The transportation of Ca^2+^ from roots to aboveground organs mainly relies on plant transpiration [4]. Peanut pods are hardly able to absorb Ca^2+^ transported from roots or other aboveground organs through xylem [5]. Previous studies have reported that Ca^2+^ deficiency in soil can induce the abortion of peanut embryos or prevent kernel expansion, and this may eventually lead to the reduction of peanut yields [4, 6]. Calcium supplementation would improve pod growth and development through calcium signal transduction pathway and promote the normal transition of the gynophores to the reproductive development [6, 7]. Li et al. found that both embryo and seed testa need to absorb Ca^2+^ that’s excreted from the shell to ensure pod growth and development, and that peanut seed development was regulated by the collaboration of Ca^2+^ signal transduction and hormone regulation pathway [8]. Chen et al. identified multiple signal pathways involved in embryo abortion under calcium deficiency, which increased IAA and ethylene but decreased ABA, gibberellin, cytokinin and brassinosteroid levels [9]. Zhang et al. also found that calcium application could improve disease resistance to soil-borne pathogens by enriching them with specific dominant bacteria [10].

As the development of next-generation sequencing, RNA-Seq technology nowadays can be used in many biological studies such as single-cell gene expression, RNA translation, RNA structure and spatial transcriptomic studies [11]. Weighted co-expression network analysis (WGCNA) is an important downstream method following RNA-Seq to find modules of highly correlated genes and relating modules to external traits to unveil the regulatory mechanisms of different organs and tissues, different growing stages in the same tissue, or biological responses at different time under abiotic stresses [12]. WGCNA has been used to explain molecular mechanisms such as tomato growth and development [13], banana maturity [14], sugar accumulation in sugarcane [15] as well as peanut embryo abortion [9], in which gene modules associated with plant hormone signaling, MAPK signaling, ubiquitin mediated proteolysis, reserve substance biosynthesis and metabolism pathways were identified to decipher regulatory network involved in peanut embryo abortion under calcium deficiency [9].

Despite the global view of peanut transcriptome under calcium deficiency, previous studies only reported differentially expressed genes (DEGs) between calcium deficient and sufficient conditions in the same cultivar [4, 6, 8–10], and few of them made comparisons between different cultivars with different seed size, which showed tolerance inconsistency under calcium deficiency. Pod fullness was reported to be influenced by seed calcium concentration, which was affected by seed size, root absorption ability and soil calcium fertility during seed development stage [16]. However, no previous studies delved into the molecular mechanisms behind different cultivars to show the reasons causing tolerance inconsistency under calcium deficiency. Thus, in our study, we aim to explain the reasons for the different tolerance between large-seed (“XH2008”) and small-seed (“Lanshan”) cultivars and unveil their different molecular mechanisms responding to calcium deficiency. This study will lay a solid foundation for further research in unveiling the genes controlling tolerance to calcium deficiency and will play a crucial role in breeding for peanut cultivars with low-calcium tolerance.

## Results

### Two peanut cultivars exhibiting different stress tolerance under low calcium conditions

**Fig. 1:**
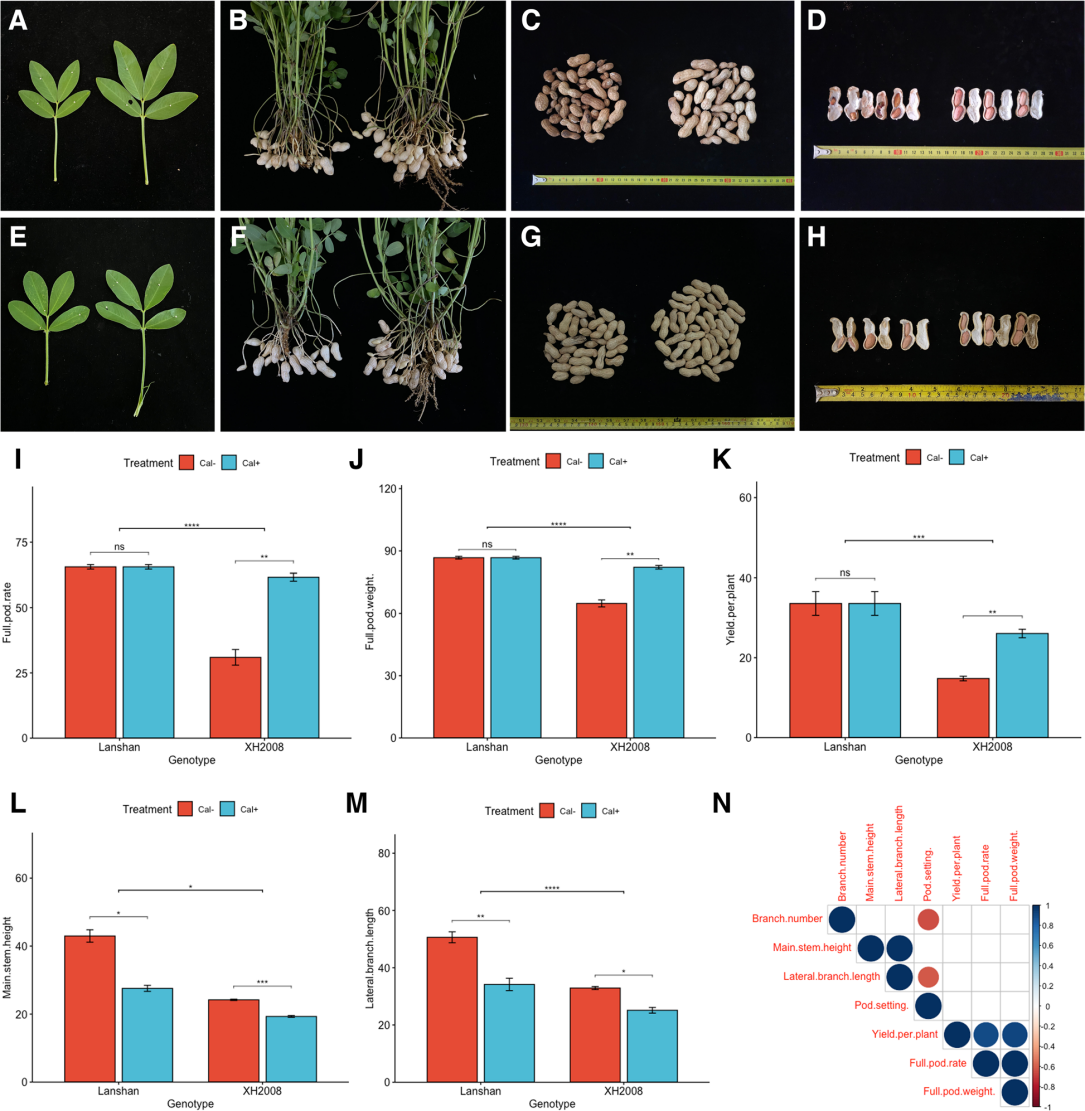
The morphological difference in leaf, pod and kernel of two cultivars under different calcium treatments. **A** the leaf morphology in “XH2008”; **B** the half-plant morphology in “XH2008”; **C** the pod morphology in “XH2008”; **D** the kernel morphology in “XH2008”. **E** the leaf morphology in “Lanshan”; **F** the half-plant morphology in “Lanshan”; **G** the pod morphology in “Lanshan”; **H** the kernel morphology in “Lanshan”; **I** The comparison between “Lanshan “ and “XH2008” in terms of full pod rate; **J** The comparison between “Lanshan “ and “XH2008” in terms of full pod weight; **K** The comparison between “Lanshan “ and “XH2008” in terms of yield per plant; **L** The comparison between “Lanshan “ and “XH2008” in terms of main stem height; **M** The comparison between “Lanshan “ and “XH2008” in terms of lateral branch number; **N** The correlation analyses among yield components and aboveground morphological characters in peanut. Note: In each sub-figure of **A**-**H**, the left corresponds to calcium deficiency and the right one corresponds to the controls; “*” represents *P* < 0.05, “**” represents *P* < 0.01, “***” represents *P* < 0.001, “****” represents *P* < 0.0001

Plant morphological and agronomic traits were evaluated to investigate the different responses in two peanut cultivars under different calcium treatments. The pot soil background information was provided in Table S1. As shown in Fig. 1A-H, there were significant differences in phenotypic changes between “Lanshan” and “XH2008” when calcium shortage occurred. In terms of pod fulfilling, “XH2008” showed high rate of empty and bad pods while the phenotypic change in economic traits was not obvious in “Lanshan”. This can also be observed in Fig. 1I-M, where full pod rate, full pod weight and yield per plant didn’t show significant changes in “Lanshan”, while “XH2008” showed significant decrease under calcium deficiency (Fig. 1I-K). As for plant architecture traits including main stem height and lateral branch length, significant decreases were observed in both cultivars after the application of calcium fertilization (Fig. 1L, M). Among all the phenotypic traits collected, only branch number neither showed significant genotypic effects nor genotypic-by-treatment interactions (Table 1). The correlation analysis among all the phenotypic traits was shown in Fig. 1N.

**Table 1:** The two-way ANOVA for seven phenotypic traits with the significance of genotypic and treatment main effects and their interactions

Phenotype	Genotype	Treatment	Genotype × Treatment
Branch Number	0.500	***	0.067
Lateral Branch Length	****	****	*
Main Stem Height	****	****	***
Full Pod Rate	****	****	****
Full Pod Weight	****	****	****
Pod Setting Rate	**	*	*
Yield per Plant	***	*	*

Genotypes included “XH2008” and “Lanshan”; Treatment levels included calcium deficiency and sufficiency levels; “*” represents *P* < 0.05, “**” represents *P* < 0.01, “***” represents *P* < 0.001, “****” represents *P* < 0.0001

### Transcriptome profiles from two peanut cultivars treated under different calcium nutrition

In order to simplify sample names, “Lanshan” (small-seed) and “XH2008” (large-seed) were simplified as “S” for small seed size and “L” for large seed size in subsequent RNA-Seq data analyses, respectively. To briefly summarize, a total of approximately 547.94 million raw reads were generated from 12 cDNA libraries by RNA-Seq. After deleting adapter sequences as well as filtering low-quality and N-containing reads, 100% raw reads were confirmed as clean reads (Table S2). A total of 547 million clean reads were filtered. The percentage of clean reads mapped to the reference genome arahy.Tifrunner.gnm1.KYV3 ranged from 75.93 to 82.75% (Table S2), suggesting that the transcriptome sequencing quality was sufficient for further analyses.

### Differentially expressed genes (DEG) analysis, enrichment analysis and qRT-PCR validation

**Fig. 2:**
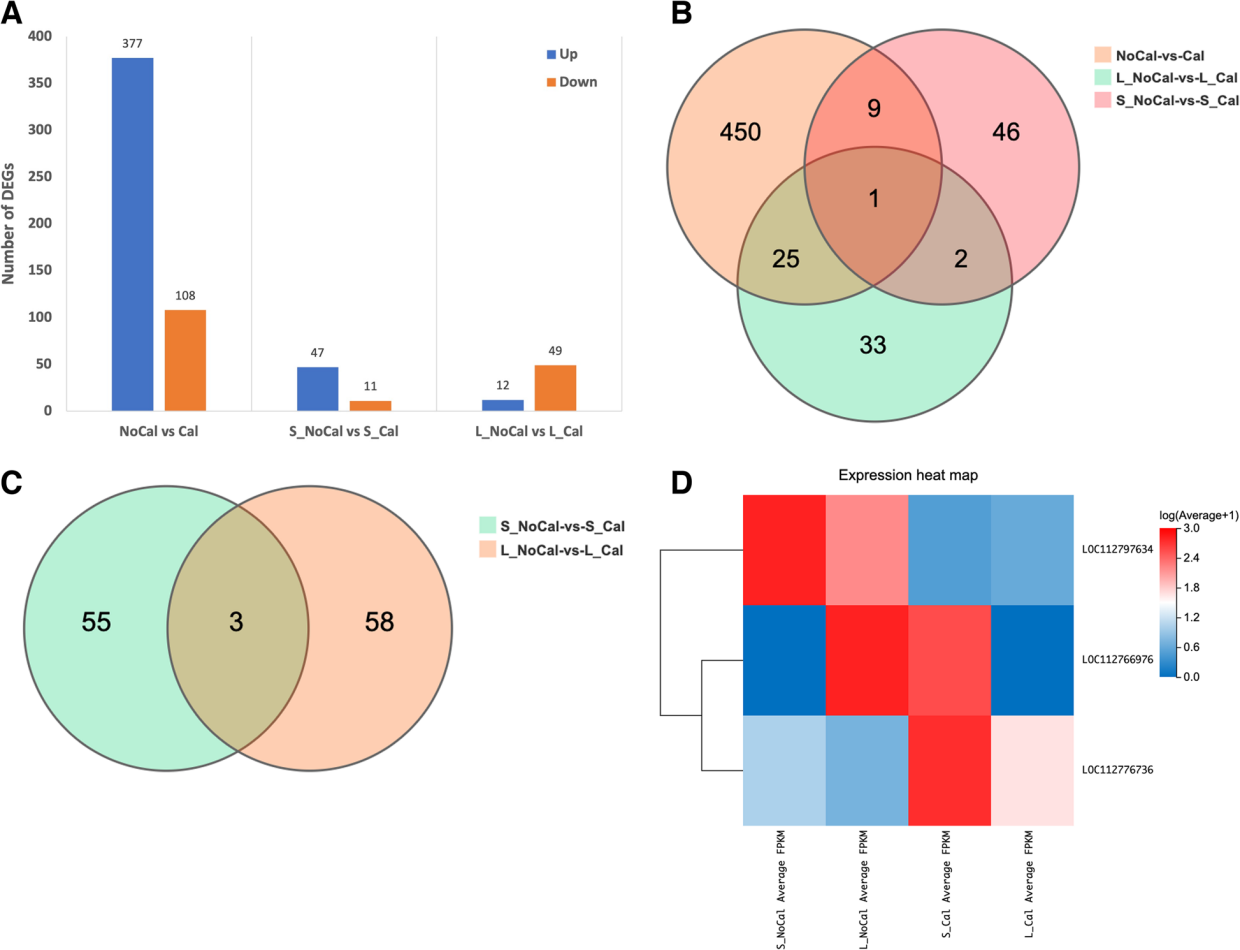
Differentially expressed genes (DEGs) analyses among six groups under different calcium treatments. **A** Number of up- and down-regulated DEGs in NoCal-vs-Cal, S_NoCal-vs-S_Cal, and L_NoCal-vs-L_Cal groups; **B** The Venn plot showed common DEGs among NoCal-vs-Cal, S_NoCal-vs-S_Cal, and L_NoCal-vs-L_Cal groups; **C** The Venn plot showing common DEGs between S_NoCal-vs-S_Cal and L_NoCal-vs-L_Cal groups. **D** The heatmap of three common DEGs between S_NoCal-vs-S_Cal and L_NoCal-vs-L_Cal groups. Note: NoCal = calcium deficiency; Cal = calcium sufficiency (CK); S_NoCal = small-seed cultivar (“Lanshan”) under calcium deficiency; S_Cal = small-seed cultivar (“Lanshan”) under calcium sufficiency; L_NoCal = large-seed cultivar (“XH2008”) under calcium deficiency; L_Cal = large-seed cultivar (“XH2008”) under calcium sufficiency

In general, a threshold of |log_2_FC|≥ 2 and FDR < 0.05 were used to screen out differentially expressed genes (DEGs). The number of DEGs after filtration were 485, 58 and 61 in three groups (NoCal vs Cal, S-NoCal vs S-Cal, and L-NoCal vs L-Cal), respectively (Fig. 2A). Of these DEGs between NoCal and Cal, 377 were up-regulated and 108 were down-regulated (Fig. 2A). Figure 2A identified 47 up-regulated and 11 down-regulated DEGs in “Lanshan” while 12 up-regulated and 49 down-regulated DEGs in “XH2008” under different calcium treatments. The Venn plots suggested that only one gene (LOC112797634) was shared among the three comparison groups (Fig. 2B). Only 3 common DEGs were shared between two cultivars (Fig. 2C), and the gene expression heatmap was shown in Fig. 2D. Among these 3 DEGs, LOC112766976 was uncharacterized with no function annotation. LOC112776736 and LOC112797634 were annotated as phosphoenolpyruvate carboxylase kinase 1 and UDP-glycosyltransferase 74G1, respectively. Phosphoenolpyruvate carboxylase kinase 1 belongs to a calcium-independent kinase, which is involved in light-dependent phosphoenolpyruvate carboxylase phosphorylation. UDP-glycosyltransferase 74G1 is involved in steviol glycoside biosynthesis.

**Fig. 3:**
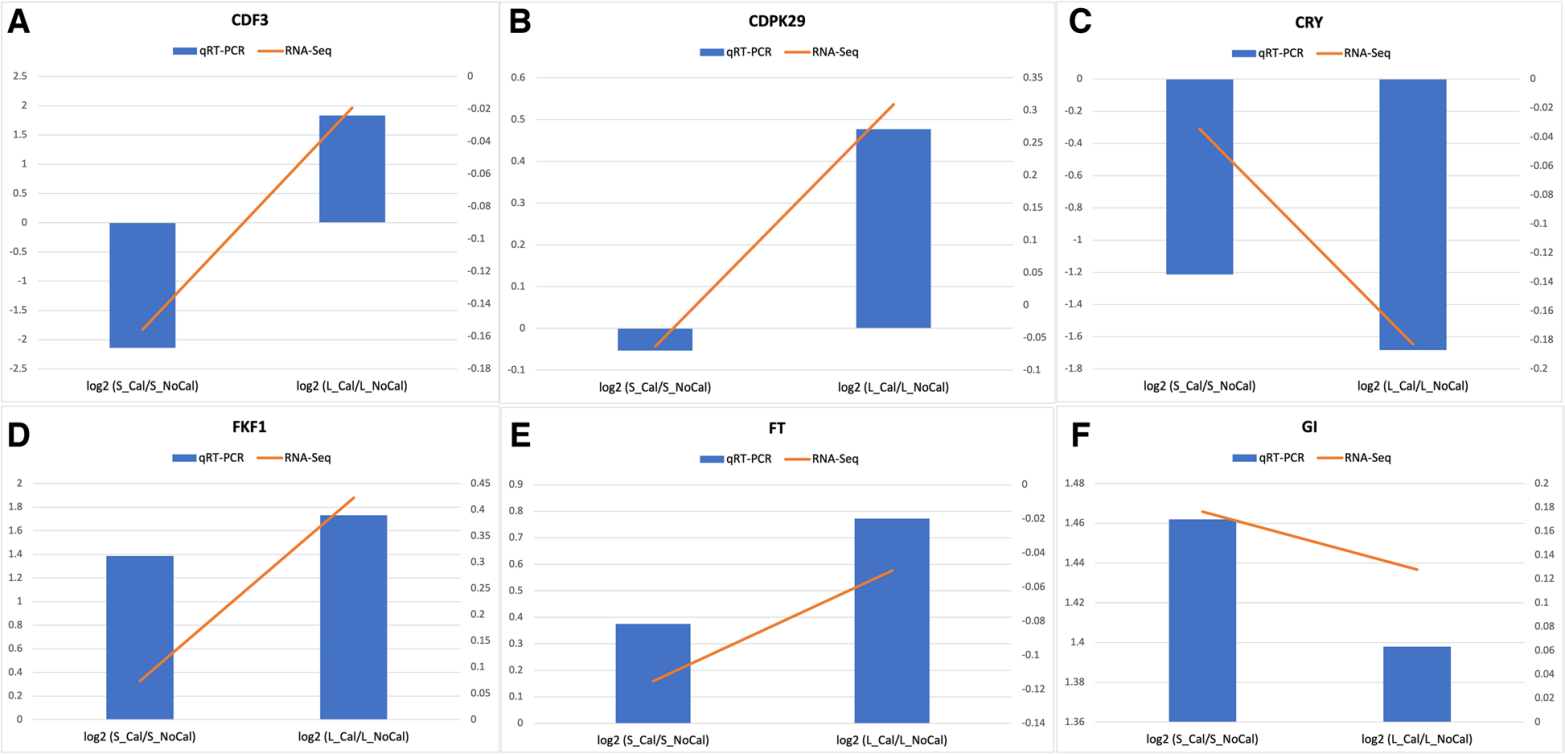
The validation of RNA-Seq results using qRT-PCR in six genes. Note: CDF3: cyclic dof factor 3 (LOC112708248); CDPK29: calcium-dependent protein kinase 29 (LOC112791189); CRY: cryptochrome-1-like (LOC112775981); FKF1: adagio protein 3-like (LOC112792033); FT: FLOWERING LOCUS T-like (LOC112698242); GI: protein GIGANTEA-like (LOC112712475)

To experimentally validate the reliability of RNA-Seq data, six genes were selected to perform qRT-PCR (Table S3). As shown in Fig. 3, the selected genes had consistent expression patterns between RNA-Seq and qRT-PCR, confirming that the RNA-seq result was reliable.

### Co-expression network analysis identified key modules correlated with low-calcium tolerance

**Fig. 4:**
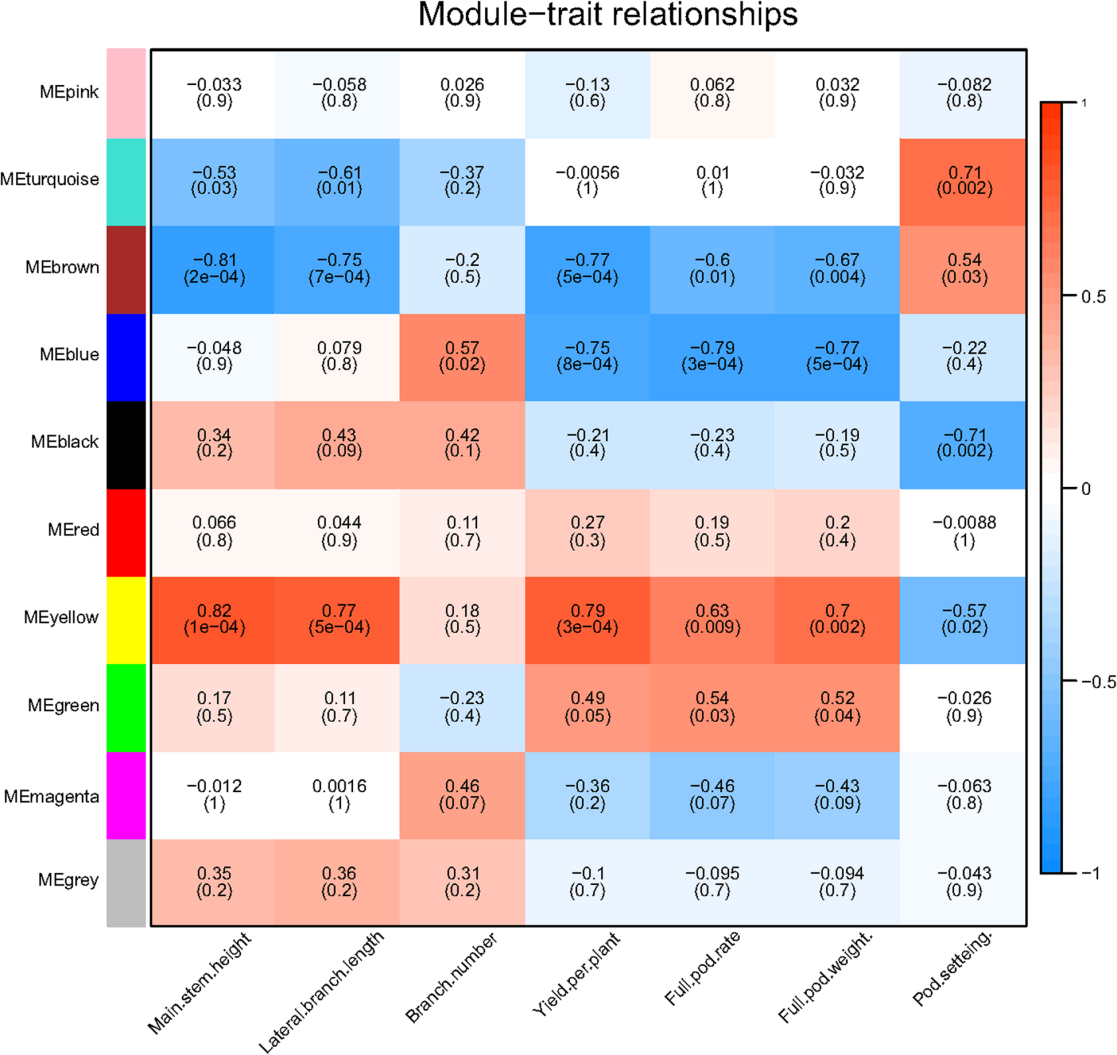
Module-trait relationships in weighted co-expression network analysis (WGCNA) for two peanut cultivars showing the relationships between gene modules and phenotypic traits

To identify the gene expression related to calcium deficiency in two peanut cultivars, a gene co-expression network was constructed using WGCNA. A total of 2,650 genes were selected and assigned to ten co-expression gene modules (Figure S1). As shown in Figure S2, we successfully identified two modules significantly associated with two peanut cultivars under different calcium treatments. The green module (MEgreen) was only positively correlated with “Lanshan” under calcium sufficiency, while the blue module (MEblue) was only positively correlated with “XH2008” under calcium deficiency. Thus, the MEgreen and MEblue modules were specifically identified in small- and large-seed cultivars under calcium deficiency, respectively. Even though brown and yellow modules showed differences between two cultivars, the changes were not found between different calcium treatments within each cultivar, and these two modules were thus not further discussed in the current study. In order to connect module genes with phenotypic traits, a module-trait relationship analysis was performed using module eigengenes and the phenotypic data (Table S4). As shown in Fig. 4, the MEgreen module was positively correlated with full pod rate (*r* = 0.54, *p* = 0.03) and full pod weight (*r* = 0.52, *p* = 0.04). In contrast, the MEblue module had a significant negative correlation with yield per plant (*r* = -0.75, *p* = 8e-04), full pod rate (*r* = -0.79, *p* = 3e-04), and full pod weight (*r *= -0.77, *p* = 5e-04), while positively correlated with branch number (*r* = 0.57, *p* = 0.02).

### GO and KEGG enrichment analysis of the key modules

**Fig. 5:**
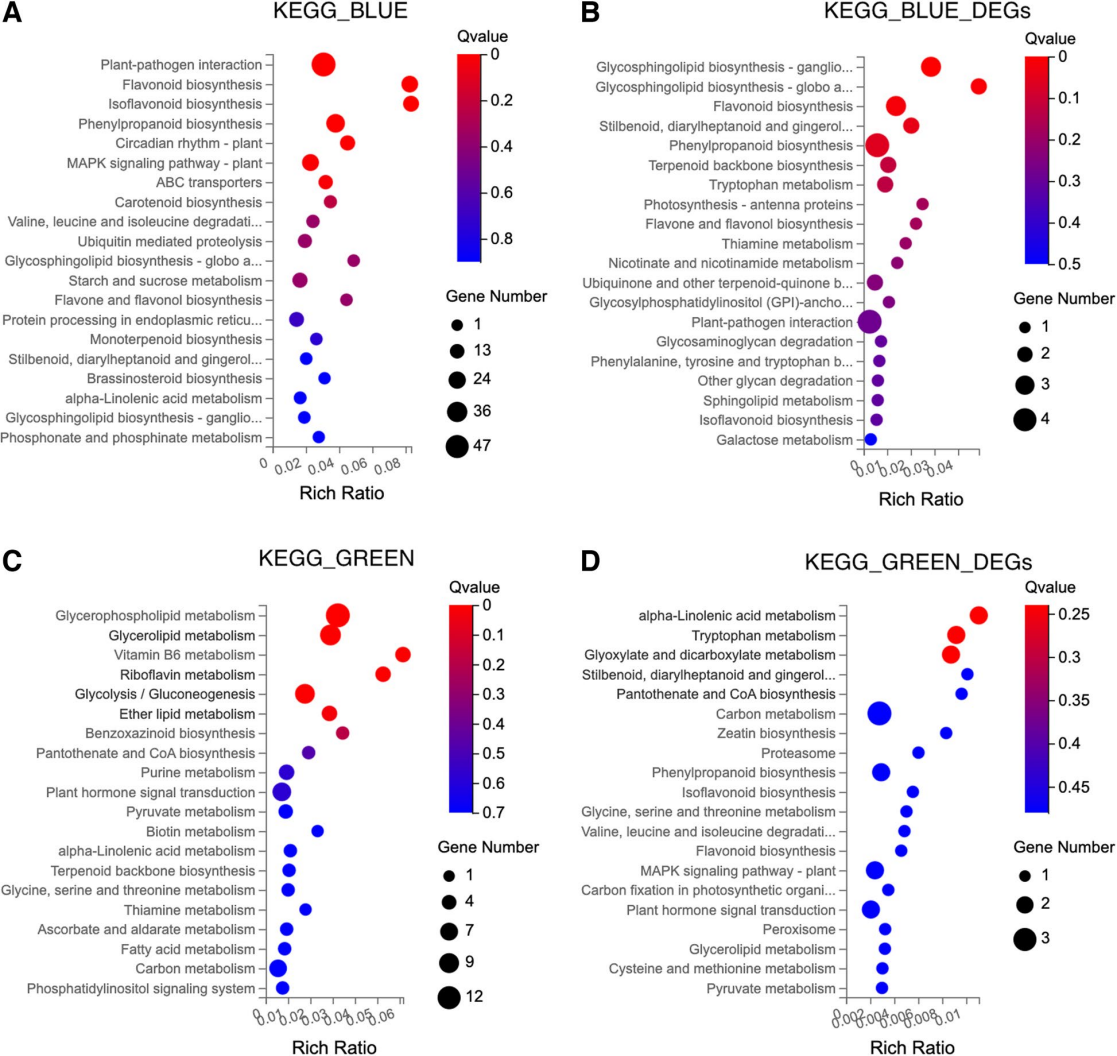
KEGG enrichment analysis for blue and green modules and their DEGs. **A** KEGG pathway enrichment analysis for the blue module; **B**) KEGG pathway enrichment analysis for the DEGs within the blue module; **C**) KEGG pathway enrichment analysis for the green module; **D**) KEGG pathway enrichment analysis for the DEGs within the green module

KEGG analysis was performed in the 514 genes of the MEblue module (Fig. 5A). Multiple crucial pathways involved specifically in “XH2008” were identified, including “plant-pathogen interaction”, “flavonoid biosynthesis”,

“isoflavonoid biosynthesis”, “phenylpropanoid biosynthesis”, “circadian rhythm”, “MAPK signaling pathway” and “ABC transporters”. All of the 61 DEGs between L-NoCal and L-Cal groups were covered by MEblue module and were further subjected for KEGG enrichment analysis. The major KEGG pathways enriched by these 61 DEGs were “glycosphingolipid biosynthesis” and “flavonoid biosynthesis” (Fig. 5B & Table S5). Within the MEgreen module, we screened 239 genes to perform KEGG analysis (Fig. 5C). Multiple crucial pathways such as “glycerophospholipid metabolism”, “glycerolipid metabolism”, “vitamin B6 metabolism”, “riboflavin metabolism”, “glycolysis/gluconeogenesis” and “ether lipid metabolism” were involved specifically in “Lanshan”. All of the 58 DEGs between S-NoCal and S-Cal groups were covered by MEgreen module and were further subjected in KEGG enrichment analysis. The major KEGG pathways enriched by these 58 DEGs were involved in “alpha-linolenic acid metabolism”, “tryptophan metabolism”, and “glyoxylate and dicarboxylate metabolism” (Fig. 5D & Table S6).

**Fig. 6:**
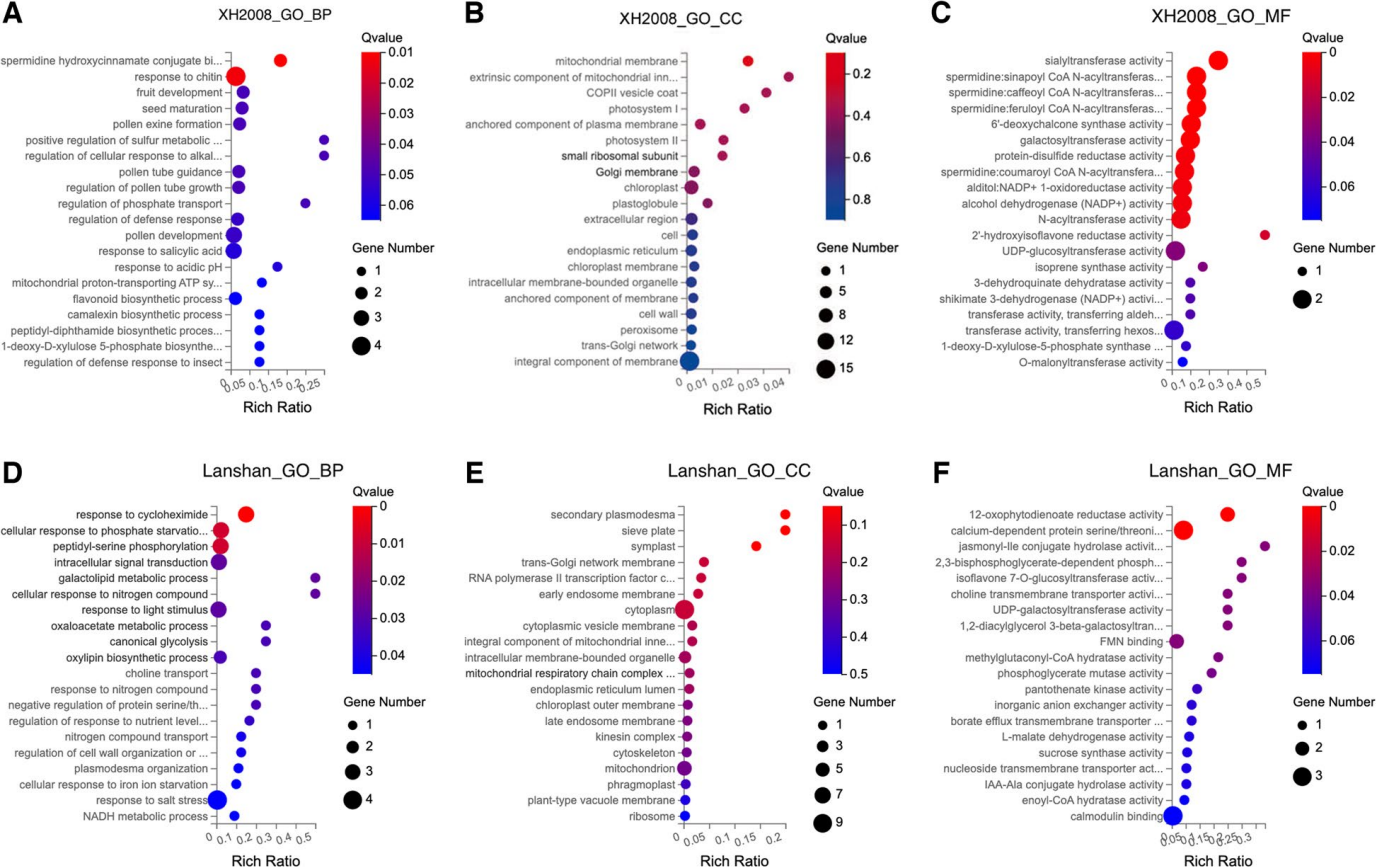
GO term analysis for DEGs in “XH2008” and “Lanshan”. **A** the biological process enriched in the DEGs of “XH2008”; **B** the cellular component enriched in the DEGs of “XH2008”; **C** the molecular function enriched in the DEGs of “XH2008”; **D** the biological process enriched in the DEGs of “Lanshan”; **E** the cellular component enriched in the DEGs of “Lanshan”; **F** the molecular function enriched in the DEGs of “Lanshan”

GO enrichment analyses were also conducted in these DEGs. Figure 6A-C and Fig. 6D-F showed GO term enrichment results of DEGs in “XH2008” and “Lanshan”, respectively. In terms of cellular component, “XH2008” was mainly involved in mitochondria and photosystem, while “Lanshan” mainly involved in secondary plasmodesma, sieve plate and symplast. In terms of biological process, “XH2008” was mainly enriched in spermidine hydroxycinnamate conjugate biosynthetic process, response to chitin, fruit development and seed maturation, while “Lanshan” was mainly enriched in response to cycloheximide, cellular response to phosphate starvation, peptidyl-serine phosphorylation and intracellular signal transduction.

### Hub genes involved in calcium deficiency tolerance via WGCNA

**Fig. 7:**
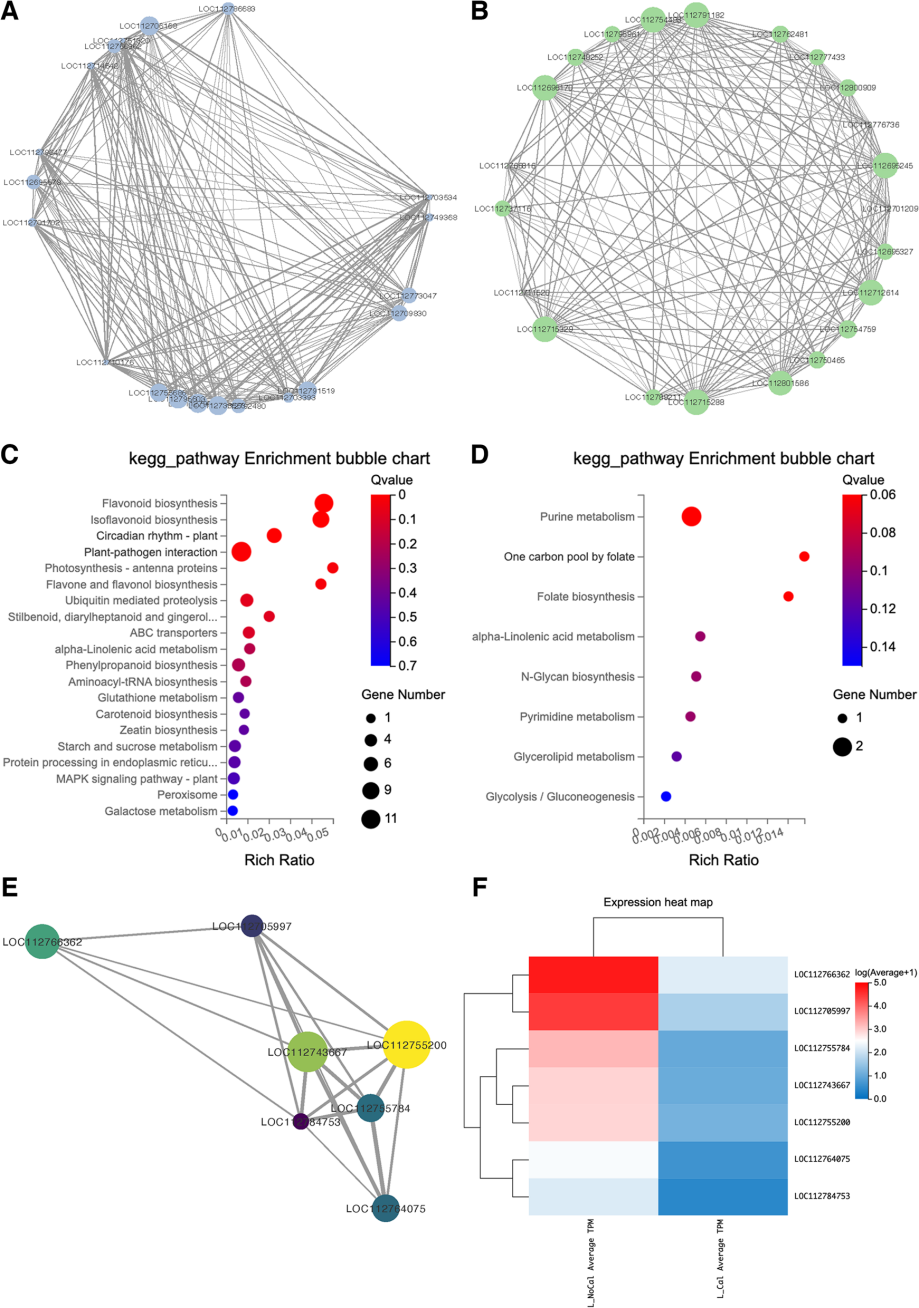
The connectivity of hub-genes identified from blue and green modules. **A** the top 20 hub-genes in the blue module of “XH2008”; **B** the top 20 hub-genes in the green module of “Lanshan”; **C** KEGG enrichment analysis of hub-gene sets within blue module; **D** KEGG enrichment analysis of hub-gene sets within green module; **E** the relationship of hub-DEGs in “XH2008”; **F** the heatmap of seven hub-DEGs identified in “XH2008” under different calcium conditions

The key genes associated with low-calcium tolerance in blue and green modules were selected using WGCNA. As a result, a total of 128 and 35 genes were selected as hub-gene sets from the blue and green modules, respectively. Based on the connection degree of each node in the gene network analysis, top 20 genes with connectivity degree larger than 97 and 17 were regarded as “hub genes” in blue and green modules, respectively (Fig. 7A-B). KEGG enrichment analyses were separately conducted for the two hub-gene sets (Fig. 7C, D). Gene annotation and network analysis information of the hub-gene sets were provided in Tables S7 & S8.

**Fig. 8:**
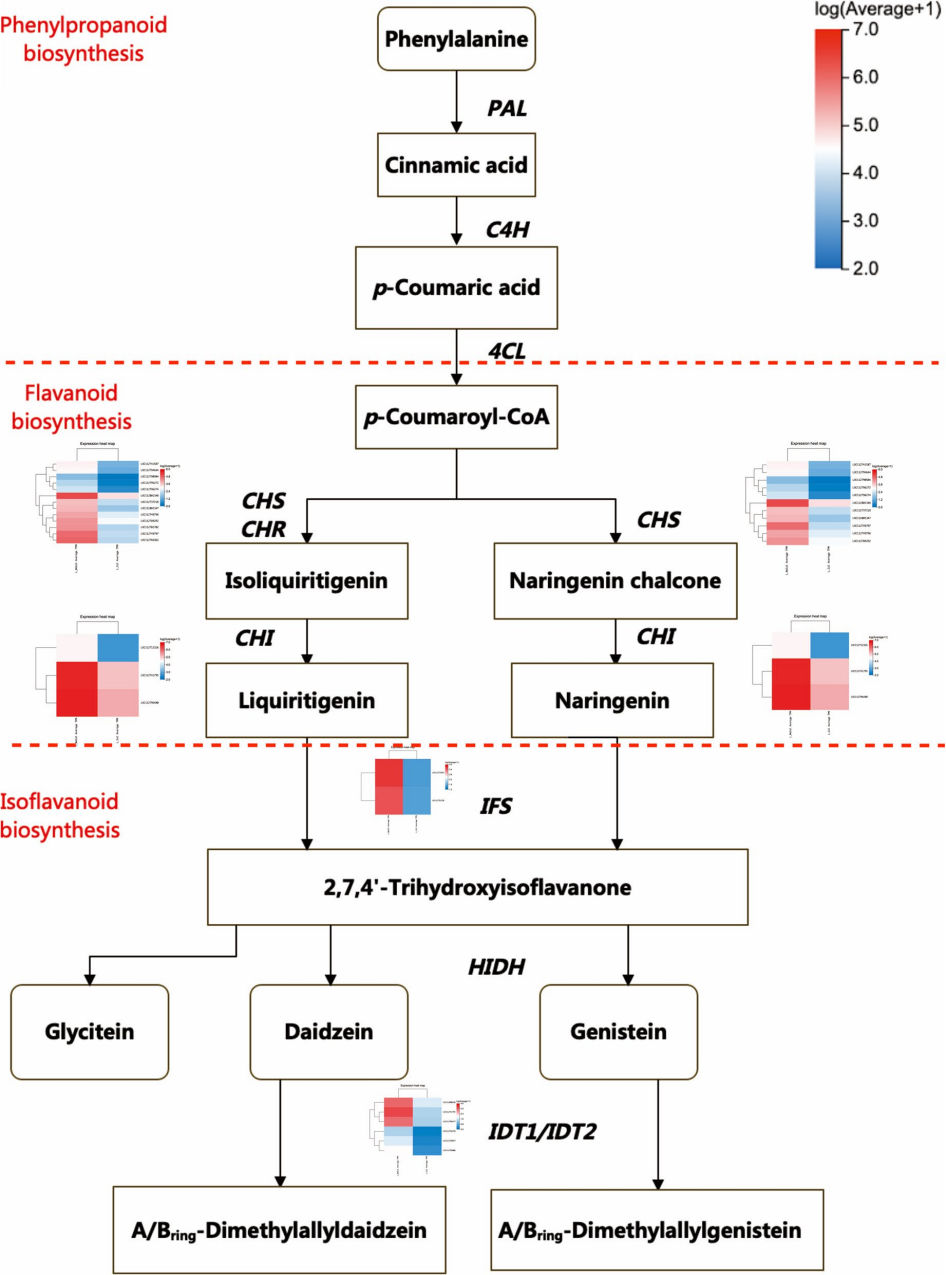
The gene expression profiling alongside the biosynthesis pathway of phenolic compounds. *PAL*: phenylalanine ammonia-lyase; *C4H*: trans-cinnamate 4-monooxygenase; *4CL*：4-coumarate-CoA ligase; *CHS*: chalcone synthase; *CHR*: chalcone reductase; *CHI*: chalcone isomerase; *IFS*: 2-hydroxyisoflavanone synthase; *HIDH*: 2-hydroxyisoflavanone dehydratase; *IDT1/IDT2*: isoflavone dimethylallyltransferase 1/ isoflavone dimethylallyltransferase 2. Note: the KEGG pathways were retrieved from the Kanehisa Laboratories [17]

Among these hub-gene sets, 7 and 1 hub genes were identified as differentially expressed hub genes (hub-DEGs) within the blue and green module, respectively (Fig. 7E & Table 2). Among the seven hub-DEGs identified in blue module, LOC112755784 and LOC112755200 were characterized as probably WRKY transcription factors 40 and 33, respectively, which encode WRKY DNA-binding domains and play key roles in plant-pathogen interaction as well as mitogen-activated protein kinase (MAPK) signaling pathway. MAPK signaling pathway is known as sensing the extracellular stimuli and conduct signaling transduction process. LOC112766362 and LOC112764075 were characterized as NAD(P)H-dependent 6'-deoxychalcone synthase, belonging to keto reductase family, and related to flavonoid biosynthesis pathway. LOC112743667 encodes malonyl-CoA:anthocyanidin 5-O-glucoside-6''-O-malonyltransferase, which is related to isoflavonoid biosynthesis pathway. LOC112784753 was annotated as UDP-glycosyltransferase 83A1-like, belonging to UDP-glucoronosyl and UDP-glucosyl transferase family. The differential expression pattern of these 7 hub genes was shown in Fig. 7F. The molecular mechanisms related to the generation of secondary metabolites in “XH2008” was proposed (Fig. 8) and the gene expression modes in the relevant pathways were provided in Table S9. The only one hub-DEG in green module was LOC112776736, which encodes phosphoenolpyruvate carboxylase kinase 1, which is a Ca^2+^-independent enzyme activated by a process involving protein synthesis in response to a range of signals in different plant tissues.

**Table 2:** Gene annotation and KEGG pathway for hub-DEGs in blue and green modules

Gene ID	Module	GeneBank Annotation	KEGG Pathway
LOC112784753	blue	UDP-glycosyltransferase 83A1-like	NA
LOC112766362	blue	NAD(P)H-dependent 6'-deoxychalcone synthase	Flavonoid biosynthesis
LOC112764075	blue	NAD(P)H-dependent 6'-deoxychalcone synthase	NA
LOC112755784	blue	Probable WRKY transcription factor 40	Plant-pathogen interaction
LOC112755200	blue	Probable WRKY transcription factor 33	MAPK signaling pathway; Plant-pathogen interaction
LOC112743667	blue	Malonyl-CoA:anthocyanidin 5-O-glucoside-6''-O-malonyltransferase	Isoflavonoid biosynthesis; Flavone and flavonol biosynthesis
LOC112705997	blue	Uncharacterized	NA
LOC112776736	green	Phosphoenolpyruvate carboxylase kinase 1	NA

## Discussion

In preliminary research, we found that large-seed peanut cultivars showed less tolerance while small-seed peanuts conferred higher tolerance under calcium deficiency. However, few studies compared and unveiled the differences in molecular mechanisms between two types of peanut cultivars. The purpose of this work was to explore such differences in two cultivars under calcium deficiency.

### Large-seed cultivar was less tolerant under calcium deficiency

Figure 1A-H suggested that “XH2008” (large-seed cultivar) was more sensitive to calcium shortage. More empty and bad pods occurred in “XH2008” than “Lanshan” (small-seed cultivar) when harvested under calcium deficiency, resulting in reduced full pod rate, full pod weight and thus, decreased yield per plant (Fig. 1I-K). Complementing calcium fertilization largely improved the yield in “XH2008” while no significant change was observed in “Lanshan” cultivar. Additionally, the application of calcium fertilization decreased main stem height and lateral branch length but improving pot-setting rate (Fig. 1L, M), suggesting an inhibiting effect of calcium fertilizer on the vegetative development. Such negative correlation (Fig. 1N) may also represent competition relationship between vegetative and reproductive development for nutrition and energy, which was commonly observed in previous relevant studies [18, 19].

### Defensive responses and antioxidant activities were triggered in large-seed cultivar under calcium deficiency

The WRKY gene family is a class of transcription factor (TF) family playing important roles under both biotic and abiotic stresses. The WRKY TFs regulate plant defensive response to various stresses by auto- and cross-regulation with different genes and TFs [20]. Under biotic stresses, the WRKY TFs are triggered following salicylic acid (SA), jasmonic acid (JA) and ethylene (EI) mediated signaling pathways upon pathogen attack [20–23]. Under abiotic stress, the WRKY TFs are also induced and trigger a network of signaling cascades to improve stress tolerance [20, 24, 25]. In addition, the WRKY TFs are involved in plant hormone signal transduction and mitogen-activated protein kinase (MAPK) signaling cascade when exposed to stress conditions [20, 26, 27]. In agreement with this, our study showed that “plant-pathogen interaction” and “MAPK signaling pathway” were among the top KEGG pathways enriched in blue module genes (Fig. 5A), where seven differentially expressed hub genes (hub-DEGs) were identified (Table 2). Among these hub-DEGs, *WRKY33* (LOC112755200) and *WRKY40* (LOC112755784) showed higher expression under calcium deficiency in “XH2008” (Fig. 7F), suggesting that calcium deficiency might either trigger defensive response or compromised disease resistance so that the sensitive cultivar was more vulnerable to pathogen attack.

Phenolic compounds are common secondary metabolites and can be classified into different groups based on the number of carbons in the molecules. Flavonoids and isoflavonoids are groups containing C_6_-C_3_-C_6_ structure with two phenolic cycles [28]. These phenolic compounds are derivatives from phenylpropanoid pathway which is known to perform antibacterial, antifungal, anti-inflammatory, anti-cancerous and antioxidant effects [29]. Previous studies have reported that an increase in phenylpropanoid metabolism and accumulation of phenolic compounds can be observed under various environmental stresses [28, 30, 31]. Specifically, flavonoids and isoflavonoids were induced when plants are exposed to infection, coldness, low nutrient conditions, salt, drought, heat, UV radiation, and metal stress condition to mitigate the adverse effects via scavenging ROS [7, 30–33]. Likewise, our study suggested that the blue-module genes in “XH2008” were mainly enriched in flavonoid, isoflavonid and phenylpropanoid metabolism (Fig. 6A), which included the hub-DEGs such as NAD(P)H-dependent 6'-deoxychalcone synthase (LOC112766362) and malonyl-CoA:anthocyanidin 5-O-glucoside-6''-O-malonyltransferase (LOC112743667), suggesting that antioxidant activities in large-seed cultivar were triggered under calcium deficiency (Table 1).

### Lipid metabolism were mainly observed in small-seed cultivar under calcium deficiency

Abiotic stress was also known to impair normal membrane function by adversely affecting fluidity, stability, and permeability [34]. Relevant to this, plants have evolved lipid metabolism to maintain membrane fluidity, stability, and permeability, thus maintaining normal cellular homeostasis under environmental stresses. Glycerolipids including galactolipids (glycosyldiacylglycerols) and phospholipids are the major structural components to maintain cellular homeostasis during plant growth and under abiotic stresses [35]. Galactolipids mainly occur in photosynthetic membranes to ensure normal photosynthesis [36] and are also crucial to maintain chloroplast morphology, thylakoid assembly and development as well as JA synthesis, thus improves plant survival under stress [36]. Phospholipids are a class of lipids present in extraplastidic membranes and serve as substrates for the signaling transduction under environmental stress [37]. Previous studies have reported that heat stress modified and activated the enzymatic biosynthesis and metabolism of signaling lipids including glycerolipids and glycerophospholipids [34]. Similarly, our study suggested that the KEGG pathways enriched in the green module genes of “Lanshan” were mainly related to lipid metabolism such as glycerolipid, glycerophospholipid, and alpha-linolenic acid metabolisms (Fig. 5C), suggesting that the tolerant cultivar concentrated more on maintaining normal photosynthetic capability and signal transduction when compared with the sensitive large-seed cultivar, which mainly triggered defensive responses and antioxidant activities under calcium deficiency.

### Proposed molecular response in the sensitive large-seed cultivar

Abiotic stress usually triggers the excessive accumulation of reactive oxygen species (ROS), which causes oxidative stress that leads to protein denaturation, lipid peroxidation, and nucleotide degradation, resulting in cellular damage and even cell death [38]. Plants have evolved to deal with oxidative stress via an endogenous defensive mechanism consisting of different enzymatic and non-enzymatic antioxidants [38]. Phenolics are a class of non-enzymatic antioxidants that are synthesized through the phenylpropanoid pathway. As universally present secondary metabolites in plants, phenolic compounds have been reported to protect plants against various biotic and abiotic stresses such as pathogen and insect attack [39], coldness [32], salt [7] and heat [7] via scavenging ROS and mitigating the adverse effects induced by oxidative stresses [28, 40, 41]. Figure 8 showed the relevant biosynthesis pathways. The biosynthesis of phenolic compounds starts from phenylalanine in phenylpropanoid pathway and catalyzed by a series of enzymes to generate *p*-Coumaroyl-CoA, which serves as the essential starting substrate for flavonoid pathway [30]. *p*-Coumaroyl-CoA was then catalyzed by chalcone synthase/chalcone reductase (*CHS/CHR*) and chalcone synthase (*CHS*) to synthesize isoliquiritigenin and naringenin chalcone, respectively, followed by the catalysis of chalcone isomerase (*CHI*) to form liquiritigenin and naringenin, which act as the branch point to enter the isoflavonoid pathway [42] under the catalysis of isoflavone synthase (*IFS*). IFS is the major enzyme controlling the isoflavonoid biosynthesis [40]. Daidzein, glycitein and genistein are the three isoflavone aglycones that could generate either isoflavones or prenylated isoflavonoids through prenylation procedures [40]. By further looking into the gene expression modes in the enzymes alongside the above biosynthesis pathways, we found that all the enzymes in the flavonoid and isoflavanoid pathways were upregulated under calcium deficiency (Fig. 8), which demonstrated that calcium deficiency might trigger ROS accumulation and thus needs the phenolic compounds to alleviate the adverse effects.

## Materials & methods

### Plant materials and calcium fertilization treatments

Two cultivars with different seed size were used in the experiment. The small-seed cultivar “Lanshan” showed higher tolerance when compared to large-seed cultivar “XH2008” under calcium deficiency. The tested peanut cultivars were bred by the Arid Land Crops Research Institute of Hunan Agricultural University (Changsha, Hunan). In the current study, “Lanshan” and “XH2008” were planted in large PVC basins (36 cm *75 cm *0.5 cm) with each one filled with 90 kg soil. The soil background information was provided in Table S1. The base fertilizer containing 3.857 g urea, 7.897 g potassium dihydrogen phosphate, and 17 g magnesium chloride hexahydrate was applied on the top 30 cm of soil surface in each basin to complement nitrogen (N), phosphate (P), potassium (K) and magnesium (Mg). Two cultivars were treated under different calcium fertilization levels with each treatment replicating three times. The red soil including exchangeable calcium of 1.48 cmol/kg was used as Ca^2+^ deficiency treatment while the soil fertilized with plaster (equivalent to 50 kg CaO per acre) was used as Ca^2+^ sufficiency treatment. CaO was applied before sowing. Seeds were sowed when 70% water maintained in the surface area, followed by plant growth and development in the outdoor condition. Watering and weeding management were applied uniformly across treatments. In the late flowering stage, peanut leaves were harvested, frozen in liquid nitrogen and then stored at − 80℃. Three biological replicates were used in this study. The mixed samples were used in the following RNA isolation and transcriptome sequencing.

### Phenotypic evaluation of two peanut cultivars

Plant morphological traits such as main stem height (cm), lateral branch length (cm), and branch number were recorded. Phenotypic traits related to yield components such as full pod rate (%), full pod weight (g), pod-setting %, and yield per plant (g) were also calculated. All the measuring processes were biologically repeated in three independent and parallel experiments. Student’s t-test was performed to calculate the *p*-values using R software (v 4.2.1).

### RNA-Sequencing

Total RNA was isolated from harvested samples using ethanol precipitation protocol and CTAB-PBIOZOL reagents according to the manufacturer’s instructions. Agilent 2100 and NanoDrop were used to detect RNA quality and purity. Only high-quality RNA samples were chosen for RNA-Seq analyses. mRNA libraries were constructed in BGI-Shenzhen and libraries were sequenced using the paired-end 150 (PE150) technology on the BGIseq500 platform (BGI-Shenzhen, China). In order to simplify sample names, “Lanshan” and “XH2008” during RNA-Seq data analyses were replaced with “S” for small seed size and “L” for large seed size, respectively. “NoCal” and “Cal” represented different calcium treatments.

### Reads cleaning and mapping

The quality of raw reads was firstly checked. In this step, clean reads was obtained by removing reads containing adapters, more than 10% of unknown nucleotides (N), and more than 50% of low quality (Q-value ≤ 20) bases. Meanwhile, the number of clean reads with Q20 (99% base call accuracy) and Q30 (99.9% base call accuracy) were calculated respectively. Filtered clean reads were then mapped to the reference genome sequence (GCF_003086295.2_arahy.Tifrunner.gnm1.KYV3) using the HISAT2 software (v 2.2.4) [43]. The mapped reads of each sample were assembled using StringTie (v 1.3.1) [44]. The gene expression level was normalized using the TPM (Transcripts per Million) method. All the downstream analyses were based on high-quality clean data. Dr. Tom system from BGI company was used for the following bioinformatics analyses. Whole dataset has been deposited in the NCBI Sequence Read Archive.

### Differentially expressed gene(DEG) analysis

Differential gene expression analysis of all the treatment groups was performed by DESeq2 [45] in the Dr. Tom system from BGI-Shenzhen. The false discovery rate (FDR) or q-value was used to correct *p*-values. Differentially expressed genes with the value of |log_2_FC|≥ 2 and an FDR value (q-value) ≤ 0.05 among all the comparison groups were considered to be significant.

### Weighted gene co-expression network analysis (WGCNA) and enrichment analyses

In order to satisfy the minimum sample requirements for WGCNA, the average read count for each of the treatment group was complemented as the 4^th^ replicate. The WGCNA package implemented in R (v 4.2.1) [46] was used to construct co-expression networks based on 52,991 DEGs. The varianceStabilizingTransformation function in DESeq2 was firstly used to transform read counts of each sample, followed by variance stabilization and data normalization by removing low-abundance genes of samples (> 95%). Gene expression values were then imported into WGCNA to construct co-expression modules using blockwiseModules function with default settings, except that the power is 28, networkType is signed, mergeCutHeight is 0.25, and minModuleSize is 30. Genes were clustered into 10 correlated modules. Modules of interests were selected based on module-trait relationships.

As for the DEGs within each module, the enrichment analyses including Gene Ontology (GO) and Kyoto Encyclopedia of Genes and Genomes (KEGG) pathways were functionally annotated using the Dr. Tom system from BGI-Shenzhen. GO analysis classified the DEGs into three functional categories including biological process (BP), cellular component (CC), and molecular function (MF). GO terms and KEGG pathways with FDR value (q-value) ≤ 0.05 were regarded as significantly enriched in DEGs.

Cytoscape (v3.9.1) was then used to visualize the networks of genes within the interested modules and to present biological interaction of hub genes [47]. To identify the key genes associated with low-calcium tolerance in blue and green modules, genes with gene significance (GS) > 0.2 and module membership (MM) > 0.8 were firstly filtered as hug-gene sets, followed by filtration of top 20 hub genes with largest connectivity degree in each module. Among the hub-gene sets, differentially expressed hub-genes were further explored.

### qRT-PCR analysis

Six genes with different expression profiles obtained by Illumina RNA-seq were selected for validation by qPCR. Gene-specific primers were designed by Primer premier 5.0. The Actin gene was used as housekeeping gene. Three biological and technical repetitions were used for each sample. Quantitative real-time PCR (qRT-PCR) was run on BioRad T100 (Bio-Rad Laboratories, Inc.) using SYBR Green Supermix according to the manufacturer’s instructions. The amplification program was set as follows: 95℃ for 5 min followed by 40 cycles of 95℃ for 15 s and 60℃ for 30 s. All reactions were performed with three independent biological replicates, and the expression levels calculated for each sample were based on three technical replicates. Data were analyzed using the Bio-Rad CFX96 Manager software (Bio-Rad Laboratories, Inc.). All data from qRT-PCR amplification were calculated with 2^−△△CT^ method [48].

## Conclusion

The current study explored different molecular mechanisms between two peanut cultivars with different tolerance in response to calcium deficiency. Based on the DEGs and WGCNA results, large-seed and small-seed cultivars performed differentially under stress. Specifically, large-seed cultivar was more sensitive by triggering plant defensive responses and antioxidant activities, while small-seed cultivar focused more on maintaining membrane functions via lipid metabolism to ensure normal photosynthesis and signal transduction. Limitations do exist in this study. For example, the limited number of samples for WGCNA might impair the reliability, and functional validation of hub-genes was yet to be conducted in the future after targeted hormone profiles were supplemented. However, this study took a first step to delve into low-calcium tolerance in peanut, which has long been ignored by most peanut breeders, and will lay a solid foundation for further large-scale multi-omics study, gene functional validation as well as breeding efforts for improving low-calcium tolerance in peanut cultivars.

## Supplementary Information

**Additional file 1: Figure S1.** The weighted co-expression network analysis (WGCNA) showing clustered gene modules identified for two cultivars.

**Additional file 2: Figure S2.** The relationship between gene modules and transcriptomic samples in two peanut cultivars under different calciumtreatments.

**Additional file 3: Table S1.** Soil background information. **Table S2.** RNA-Seq data summary for "XH2008" and "Lanshan" under different calcium treatments. **Table S3.** Primer sequences of six pairs of genes to be validated using qRT-PCR. **Table S4.** The module-trait correlation analysis showing pearson correlation value and p-value. **Table S5.** The KEGG pathways enriched for 61 differentially expressed genes (DEGs) in "XH2008". **Table S6.** The KEGG pathways enriched for 58 differentially expressed genes (DEGs) in "Lanshan". **Table S7.** The topological results and  annotationinformation for hub-gene sets in the blue module of "XH2008" cultivar. **Table S8.** The topological results and  annotation information for hub-gene sets in the green module of "Lanshan" cultivar. **Table S9.** The gene expression modes for the genes annotated to secondary metabolites.

## Data Availability

All data mentioned in this article and supplementary information files were provided. The transcriptomic raw data from this study were submitted to the NCBI Sequence Read Archive (SRA) under the Bioproject accession number PRJNA924004 with the SRA accession number of SRR23083638-SRR23083649. The data can be accessible through https://dataview.ncbi.nlm.nih.gov/object/PRJNA924004?reviewer=goeff3s8fj9sv156gokqmhq0b8.
