# Supplementary figures and images for: Gene co-expression network analysis identifies hub genes associated with different tolerance under calcium deficiency in two peanut cultivars
Source: BMC Genomics. 2023 Jul 27;24:421. doi: 10.1186/s12864-023-09436-9 (PMC10373417; doi:10.1186/s12864-023-09436-9)

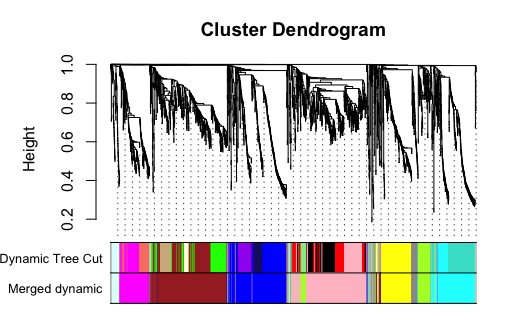

Supplement: Supplementary file 1 — Additional file 1: Figure S1. The weighted co-expression network analysis (WGCNA) showing clustered gene modules identified for two cultivars. [file 12864_2023_9436_MOESM1_ESM.tiff]

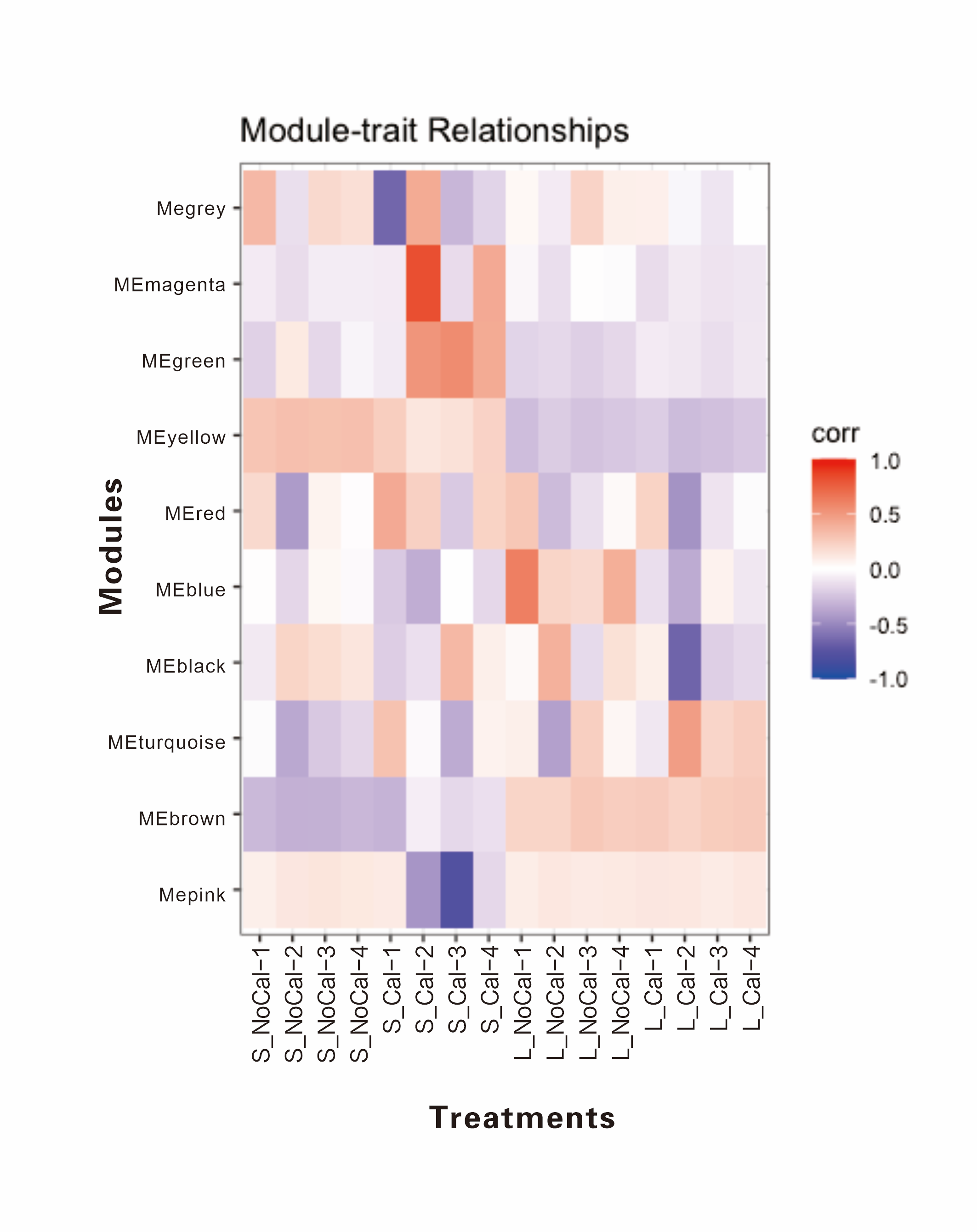

Supplement: Supplementary file 2 — Additional file 2: Figure S2. The relationship between gene modules and transcriptomic samples in two peanut cultivars under different calciumtreatments. [file 12864_2023_9436_MOESM2_ESM.tiff]
